# Illustrating changes in landscapes of passing opportunities along a set of competitive football matches

**DOI:** 10.1038/s41598-021-89184-6

**Published:** 2021-05-07

**Authors:** Luis Ignacio Gómez-Jordana, Rodrigo Amaro e Silva, João Milho, Angel Ric, Pedro Passos

**Affiliations:** 1 CIPER, Faculdade de Motricidade Humana, Universidade de Lisboa, Estrada da Costa, 1495-751 Cruz Quebrada, Portugal; 2 Instituto Dom Luiz, Faculdade de Ciências, Universidade de Lisboa, Campo grande 16, 1749-016 Lisbon, Portugal; 3 Centre for Ecology, Evolution and Environmental Changes (CE3C), Faculdade de Ciências da Universidade de Lisboa, Campo Grande, 1749‐016 Lisbon, Portugal; 4 CIMOSM, ISEL, Instituto Superior de Engenharia de Lisboa, Instituto Politécnico de Lisboa, 1959-007 Lisbon, Portugal; 5 Complex Systema in Sport Research Group, Institut Nacional D’Educacio Fisica de Catalunya (INEFC), University of Lleida (UdL), Lleida, Spain; 6 FC Barcelona, Barcelona, Spain

**Keywords:** Dynamical systems, Human behaviour

## Abstract

This study aims to illustrate the landscape of passing opportunities of a football team across a set of competitive matches. To do so positional data of 5 competitive matches was used to create polygons of pass availability. Passes were divided into three types depending on the hypothetical threat they may pose to the opposing defense (penetrative, support, and backwards passes). These categories were used to create three heatmaps per match. Moreover, the mean time of passing opportunities was calculated and compared across matches and for the three categories of passes. Due to the specificity of player’s interactive behavior, results showed heatmaps with a variety of patterns. Specifically the fifth match was very dissimilar to the other four. However, characterizing a football match in terms of passing opportunities with a single heatmap dismisses the variety of dynamics that occur throughout a match. Therefore, three temporal heatmaps over windows of 10 min were presented highlighting on-going dynamical changes in pass availability. Results also display that penetrative passes were available over shorter periods of time than backward passes that were available shorter than support passes. The results highlight the sensibility of the model to different task constrains that emerge within football matches.

## Introduction

During a football match, players adjust their decisions and actions to each other’s as they play over the hegemony of the game^1,2^ (also called dominance^3^). We assume that a team with greater hegemony will be more threatening and this threat can be characterized by how each team explores the space that is temporarily free of opponents; allowing to either keep the ball possession, progress towards the goal or create a goal scoring opportunity^4^. To do so, the players’ decisions and actions are highly influenced by prospective information that emerges in the course of the players’ interactive behavior, i.e. a temporary open gap in the opponent defense^5^. Thus, this information continuously suggests not only *what* to do, but also *when*, *where,* and *how* to do it, highlighting ongoing possibilities of action^6–8^.

This flow of prospective information is dynamically evolving over time^9^, as it is influenced by the changing positioning of the players (respective to the field boundaries, proximity to the goal, and their teammates and opponents)^6,7,10^, as well as the players’ capability to perceive what the others can do^5,7,11,12^. Thus, passing opportunities emerge, persist, and dissolve within a limited space–time window^13^, and greatly depend on the players’ interactive behavior. For instance, the interaction between the ball-carrier and the off-ball players is determinant to create passing channels at a given moment. On one hand, the ball carrier’s attunement to relevant information (e.g., relevant passing lines) influences how open an environment is for his/her teammates. On the other hand, the presence of several potential pass receivers, increases a player unpredictability regarding the actions to perform, creating additional difficulties to the opposing team^14^.The rich set of individual and environmental constraints that can arise in a football match influences the availability of a diverse set of potential actions (some menacing whereas others are not), which can be conceptualized as a landscape of potential actions^15^. Previous research addressed this issue and created a landscape of passing opportunities in football which was based on the analysis of a single match^16,17^). Results revealed the key areas on field providing most opportunities for penetrative passes^16^ and also support and backward passes^17^ on a single competitive football match. Moreover, a heterogeneous space–time spread of passing opportunities across the entire field was displayed, which highlighted the specificity of player’s interactive behaviour during the course of a match. However, these previous research works has some limitations some of them we aim to address now: (1) instead of a single match analysis, on the current research work it was analysed the dynamics of the created landscapes, within and between a set of five competitive football matches; (2) the current research work drove us to the need of analyse the images created by the heatmaps beyond visual inspection. Therefore, to allow a more objective analysis we used a technique called Earth Mover’s Distance (EMD), which quantifies the differences between the heatmaps of the five matches under analysis; (3) concerning the dynamics of the landscapes within each match, temporal heatmaps were created which allows to identify how the threat (for the defence) caused by the different types of passing opportunities changed during the course of a match.

Despite a relevant initial contribution of the previous research work, to the authors’ knowledge, there is still a gap in the literature concerning the depiction of off-ball players’ actions, and how they are affected by specific constrains (i.e. playing against different teams; possibility to receive different types of passes; changes on passing possibilities along time). Beyond the influence of these specific constraints is it possible to depict a similarity on player’s interactive behaviour along a set of matches? Aiming to answer this question, the main goal of this study was to identify the landscapes of passing opportunities as a result of spatio-temporal windows that emerge due to the interactive behaviour between off-ball players, defenders, and the ball carrier during different football matches.

## Methods

### Data acquisition

The data analyzed corresponds to five matches of the Spanish second division respective to the 2017/18 season, focusing on one team and five of its competitors. Over these matches, the team under scope had 20 participating players. The data corresponds to bi-dimensional (*x* and *y*) coordinates of each player of both teams recorded at 25 fps by an opto-tracking system. The data was recorded and provided by Footovision (Paris, France), complemented by game events (e.g., passes, shots, fouls…). This study received institutional ethics approval from Faculdade de Motricidade Humana, University of Lisbon. It is worth noting that we analyzed performance that did not require identification of individual performers.

### Algorithm description

The algorithm evaluates passing opportunities for: (1) the players’ current position and (2) to the players’ estimated position a second later (taking into account the player current velocity) as long as the velocity was higher than 2 m/s. This analysis was only carried on when the ball was in play. Based on the ball carrier and support player’s current and estimated positions, two potential passing lines were created^16^.

Afterwards, aiming to evaluate if these passing opportunities were actually available, defensive coverage areas were defined based on the defending players’ running line velocities^18^ (see^19^ for a similar approach to the one taken to create these areas). Using the velocity of each player, a matrix was created that corresponded to each turning capability in such a way that each player velocity on percentile 90 was set as the point with the lowest turning capability (40º) and a velocity of 0 m/s was stablish as the highest turning capability (360º)^18^. These turning capabilities were then used to create coverage areas (see Algorithm 1;^17^) that were used to calculate the time each defender will take to arrive to each point of the potential passing line assuming that the player will take a second to arrive to the end of the area. When the velocity of a player was under 1.5 m/s we used this value instead of the actual velocity of the player. With this, unrealistic situations were defenders did not cover any space were avoided.
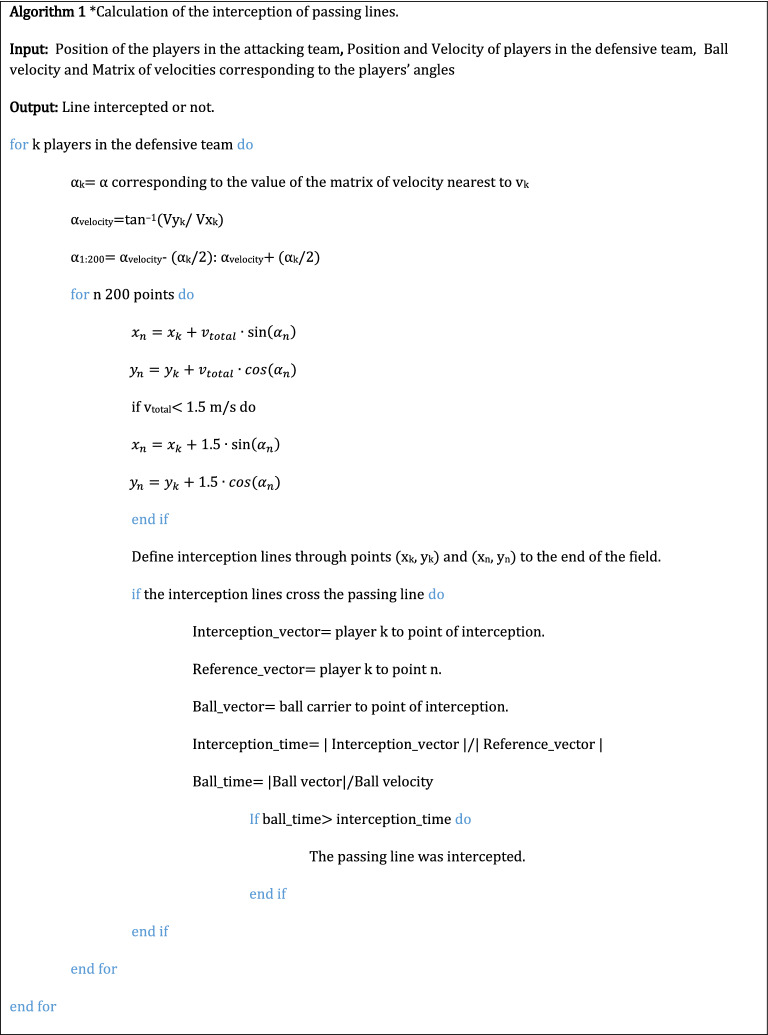


If the player could arrive to any point of the line before the ball, the pass was consider to be intercepted (see Algorithm 1). If the pass was available, a polygon was defined as the area that is temporarily available to perform a pass, with the ball carrier, the receiver and the two nearest defenders as its vertexes. In the absence of any defensive player, the sideline was used as an alternative. Based on player’s positional data this polygon was updated every 0.2 s. In this study the ball was considered to move at a constant 10 m/s speed. While this value was chosen for illustrative purposes, it is important to acknowledge that ball speed is a dynamic parameter (as it depends on the ball carrier passing ability, match context and even field conditions) and that it can, and should be, fine-tuned by the end-user.

### Pass categorization: the outplay principle

Some actions taken by attacking players in football are more threatening than others. For instance, to increase the chances to shot at goal, the ball carrier must perform passes that outplay as many opponents as possible^20^. By ‘outplay’ we mean the defending player’s that after a pass were further away from the own goal than the ball. Therefore, this *outplayed principle* is proposed in order to categorize the different types of passes that could be used to create a landscape model of passing opportunities in football. This principle expresses the number of opponents a ball carrier has in between her/him and their own goal^21,22^. Passes that outplay more opponents have been shown to be correlated with the number of goals scored^23,24^, are therefore, more threatening but are also generally more risky^3,25^.

**Figure 1:**
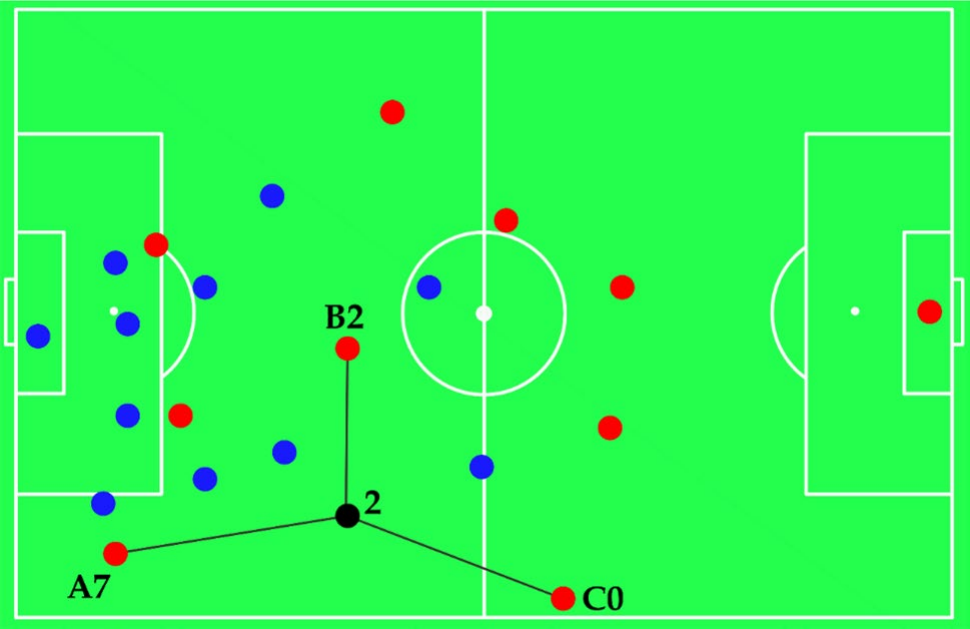
Blue dots correspond to the defensive team players, while the red dots represent the attacking team players. The black dot corresponds to the ball carrier. The numbers near each red dot (i.e., attacking player) corresponds to his/her outplay opponents. Letter A corresponds to a penetrative pass, as the attacking player (and potential receiver) outplays more opponents than the current ball carrier. Letter B corresponds to a support pass as the attacking player (and potential receiver) outplays the same number of opponents as the current ball carrier. Letter C corresponds to a backward pass as the attacking player (and potential receiver) outplays less opponents than the current ball carrier.

Hence, the passing opportunities were divided on three categories: (1) *penetrative pass,* a passing opportunity to a player that outplays more opponents than the ball carrier; (2) *support pass*, a passing opportunity to a player that outplays the same opponents than the ball carrier; and (3) *backward pass*, a passing opportunity to a teammate which outplays less opponents than the ball carrier (please see Fig. 1 for a detailed explanation).

Moreover due to the level of threat that might cause to the opposing team, it was hypothesized that different passes (based on the outplay principle^24^) may create different landscapes of passing opportunities, and due to specific task constraints that characterize each match, they could vary across different matches.

Finally, following the rationale that players are more willing to perform *penetrative* passes (as they will receive a higher reward for them) and these passes are more threatening and risky than the other two, we hypothesized that *penetrative* passes opportunities were available for shorter periods than *support* or *backward* passes.
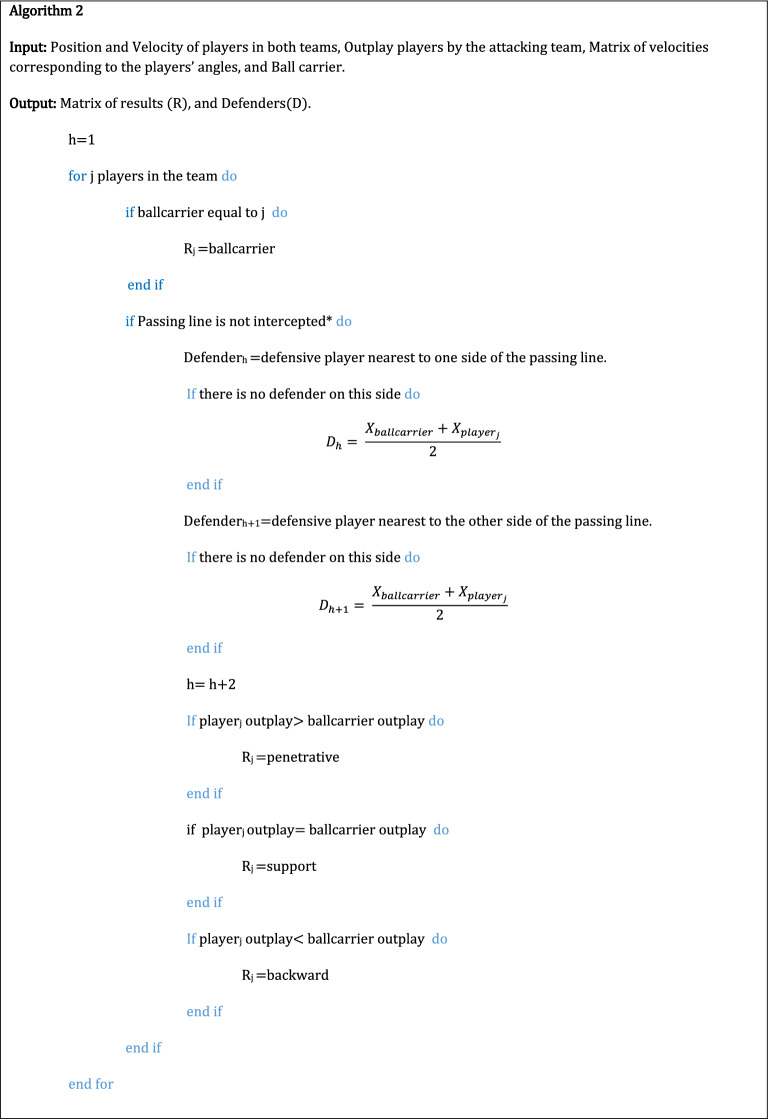


### Data repository

Additionally, we created a repository [https://github.com/luisjordana/landscape-passing-opportunities] including 3 min of data, as well as Matlab routines, including algorithms 1 and 2, and others that can be used to load and process the data, run the algorithm and create similar videos and heatmaps as the ones presented in this article.

### Data analysis

#### Landscape of passing opportunities

To define the landscape of passing opportunities, heatmaps were built by overlapping the different polygons available throughout a match. For the heatmaps to express how long a given area was under potential passing opportunities, the obtained count values were divided by the data set’s sampling rate (or frequency). Thus, and considering the color scale used, dark blue means that no passing opportunities were available, whereas dark red means that passing opportunities were available for more than 100 s per match.

A heatmap was built for each of the five matches and each of the three types of potential passes previously defined (*penetrative, support* and *backward*). To allow comparisons through the matches, the field dimensions were normalized to 105 m × 70 m.

Furthermore, to analyze the dynamics of these landscapes, heatmaps were created to characterized consecutive periods of a match. While the size of the time window and the update rate depend on the specific necessities of each team, a 10-min sliding window was chosen for illustrative purposes. For instance, for the first half of the 4th match, exemplar heatmaps of penetrative passing opportunities with time windows of 10 min were created. Three of these heatmaps are presented to show the dynamical evolution of these landscapes. On these heatmaps the color scale was adjusted to have dark red in the areas that were under potential passes for 30 s.

Additionally to go beyond a visual inspection, the heatmaps of the five matches for each type of pass were compared using the Earth Mover’s Distance (EMD; please see^26^ for further explanation of this method). This method provides a measure of similarity between two images^27^, with lower values meaning high similarity and vice versa. As an EMD output we created a colored matrix where the coldest colors (i.e., blue, green) display a high similarity between heatmaps, whereas the warmest colors (i.e., yellow, orange, red) display a low similarity between heatmaps.

#### Time of passing opportunities analysis

The time that each potential pass was available was calculated over the five matches. From this information, it is possible to calculate the mean duration of passing opportunities per match and by type of pass (please see Table 1 for further details).

**Table 1:** Mean and standard deviation of the time that the passing opportunities were available per match. The values are presented in seconds.

	1st match	2nd match	3rd match	4th match	5th match	Total
Penetrative passes	1.19 ± 1.00 s	0.97 ± 0.88 s	1.03 ± 0.97 s	0.95 ± 0.97 s	0.93 ± 1.02 s	1.00 ± 0.98 s
Support passes	1.59 ± 1.30 s	1.45 ± 1.31 s	1.54 ± 1.43 s	1.45 ± 1.29 s	1.60 ± 1.47 s	1.52 ± 1.39 s
Backward passes	1.43 ± 1.17 s	1.15 ± 0.91 s	1.10 ± 0.89 s	0.97 ± 0.78 s	1.00 ± 0.83 s	1.13 ± 0.97 s
Total	1.34 ± 1.13 s	1.15 ± 1.04 s	1.21 ± 1.18 s	1.17 ± 1.17 s	1.16 ± 1.15 s	1.26 ± 1.80 s

#### Statistical analysis

Aiming to analyze if the mean duration for passing availability differs across the different type of passes as well as across the five matches (i.e. two factors, Match x TypeOfPass), a two-way ANOVA was performed using Rstudio (Rstudio 1.138; RStudio, Inc., Boston, MA). It is important to note that since the distribution of passing availability is not normal (with most cases concentrated in shorter pass durations, but with a long tail), a requirement for properly implementing an ANOVA, a logarithmic transformation was implemented. After this transformation, q-q plots and residual plots where consistent with a normal distribution with homogeneity of variances. Furthermore, the factors were found not to be collinear.

The power of the effect is quantified by the eta squared (η^2^) statistic, with values near 0.01 meaning the effect was small, 0.06 medium, and 0.14 high^28^. To asses uncertainty the 95% confidence intervals regarding these power of the effects were provided. In case there was a significant effect of a factor and/or the interaction, a Tukey post hoc analysis was run to analyze the main effects and/or interactions between the two factors, providing information on which specific matches and/or type of passes differ. For all tests performed, the level of significance was *p* < 0.05.

## Results

### Videos

Three short videos that illustrate the landscapes of each type of passing opportunity were provided as supplementary material. The top part of Videos 1–3 displays an animation of player’s displacements during a match, as well as the polygons that were created which identifies each passing opportunity. The bottom part of Videos 1–3 displays the heatmaps that illustrates the landscapes of passing opportunities along time. These videos correspond to (1) Penetrative Passes, (2) Support Passes, and (3) Backward Passes.

### Landscapes of passing opportunities

**Figure 2:**
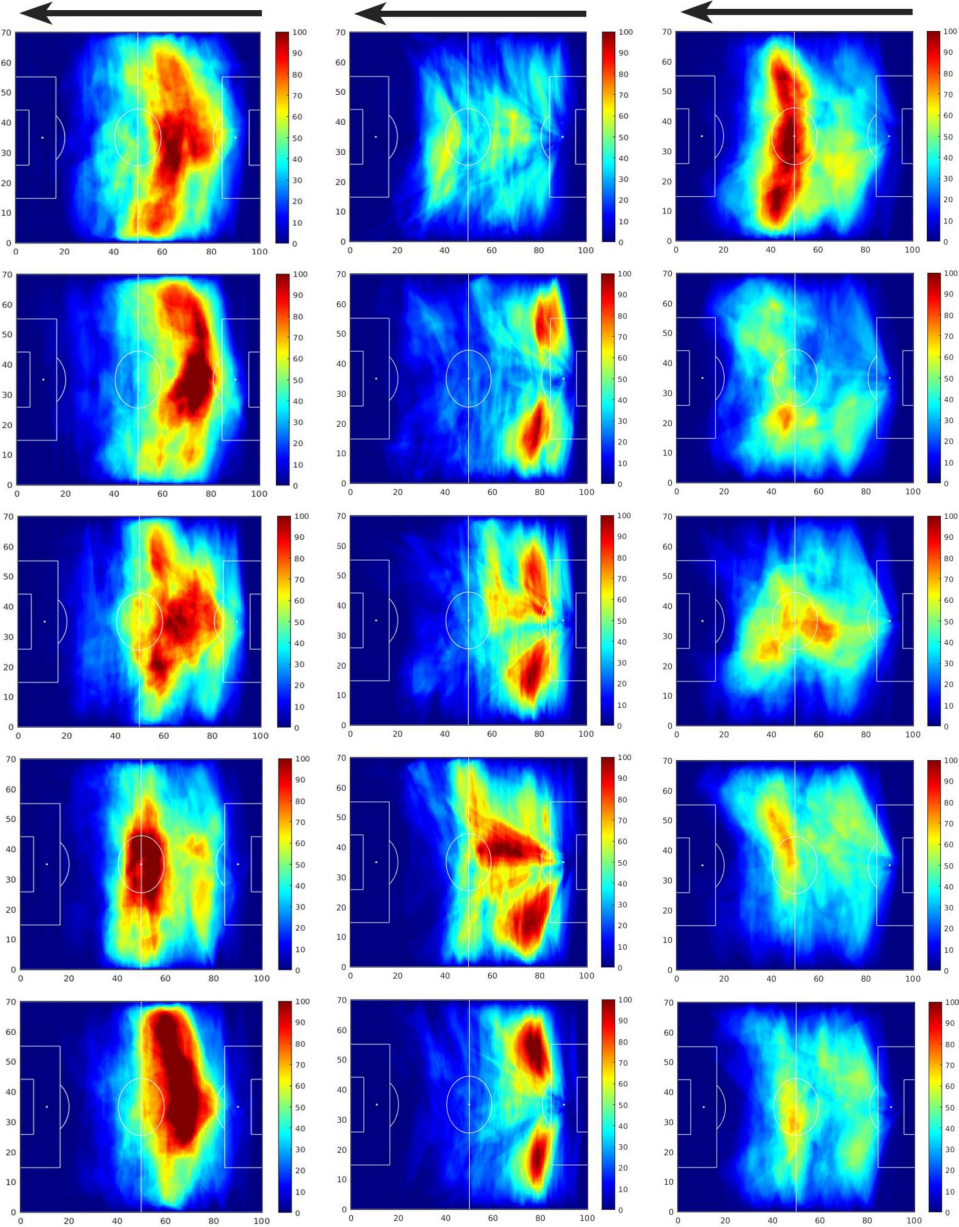
Landscape of passing opportunities for penetrative (left), support (middle), and backward (right) of the five matches. Darker blue indicates areas of the field that did not have any passing opportunities available in the course of the match, while colors nearer to dark red indicate a higher amount of passing opportunities available on that area of the field. The black arrow on the top indicates the attacker direction.

The obtained heatmaps for the five matches and three types of passes are displayed in Fig. 2. It is important to highlight the variety of landscapes that emerged over the matches. As displayed by the warmest colors (i.e., yellow, orange, red), *penetrative* (Fig. 2, left column) and *support* passing opportunities (Fig. 2, middle column) are mostly concentrated in the midfield (mainly for *penetrative* passes) and own side of the field (from the attackers perspective; mainly for *support* passes). *Backward* passing opportunities (Fig. 2, right column) are scattered more across the field and, with the exception of the first match, are not concentrated on any particular area of the field.

**Figure 3:**
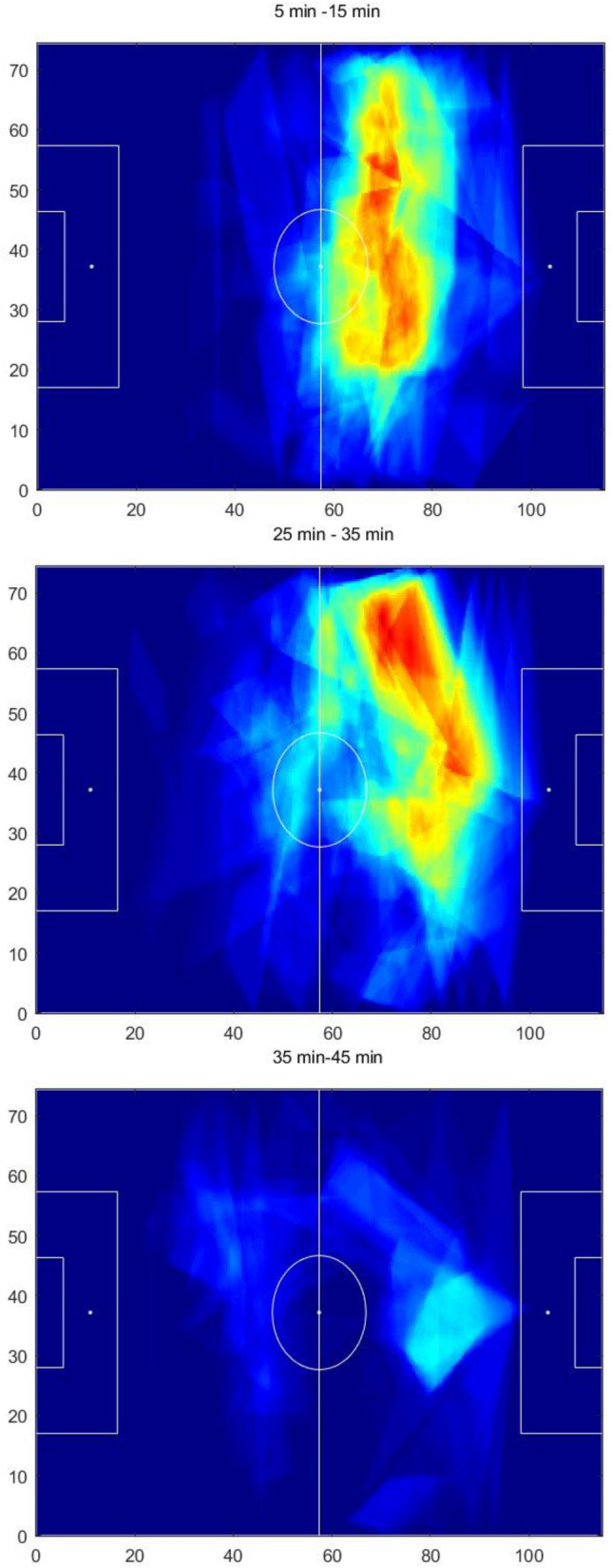
Three heatmaps of 10 min each of the first half of the 4th match representing minutes 5 through 15 (top figure), 25 through 35 (mid figure), and 35–45 (bottom figure). In these heatmaps the dark red area represents areas that were under potential passes for 30 s or more.

In Fig. 3 three 10-min windows heatmaps are shown for the 4th match which highlight how a team’s offensive dynamics fluctuate throughout a match: while between the 5th and 15th minutes opportunities were created in both the right and center regions of the field (top figure), between the 25th and 35th minute (i.e. 20 min later) diagonal penetrative passes from the center to the right were predominant as passes concentrated more in the right side of the field (center figure); lastly, in the time period from the 35th and 45th minutes we can see that pass frequency drops drastically (bottom figure).

### Heatmaps analysis with Earth Mover’s Distance (EMD)

**Figure 4:**
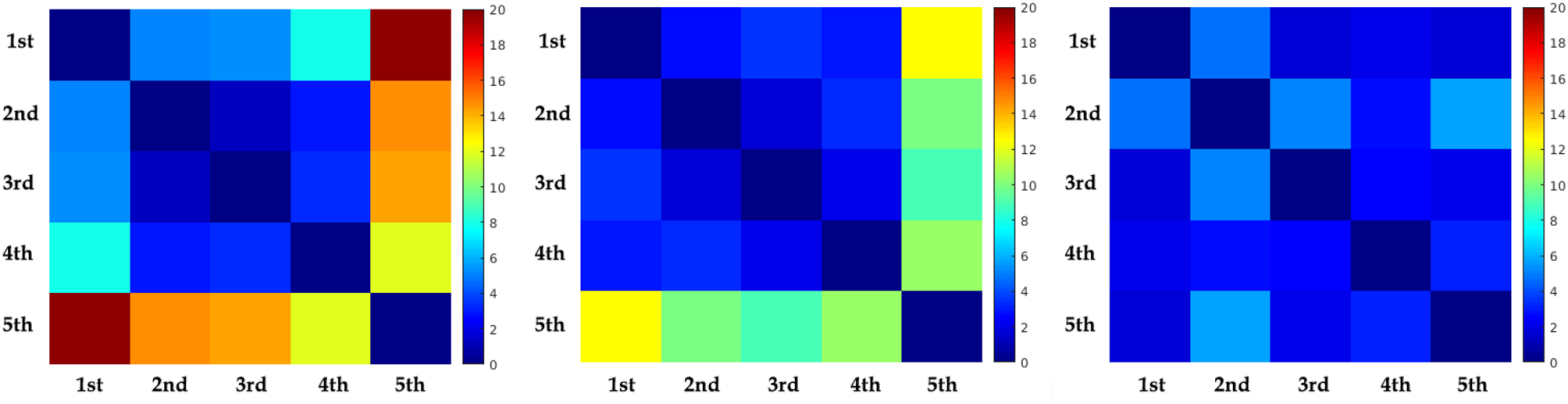
Matrix expressing values of EMD’s for Penetrative, support, and backward passing opportunities respectively. Colors nearer to color dark blue indicate more similarity in between the landscapes while colors nearer to strong red indicate more dissimilarity.

EMD results revealed that while *backward* passing opportunities (Fig. 4 on the right) are similar over the five matches, *penetrative* (Fig. 4 on the left) and *support* passing opportunities show more diverse patterns (Fig. 4 on the middle). All the squares in the *backward* passing matrix are near dark blue, meaning that the landscapes are very similar across the five matches. On the other hand, the warmest colors on the fifth match of the *penetrative* and *support* passing opportunities matrix is indicative that this fifth match is very dissimilar to the other four (please see the last line of Fig. 4 on the left and middle respectively).

### Duration of passing opportunities

The ANOVA revealed a significant effect for the factor TypeOfPass (F_(2,16132)_ = 376.80, *p* < 0.001; *Penetrative:* 1.00 ± 0.98 and *Support:* 1.52 ± 1.39, *Backward:* 1.13 ± 0.97), with a medium effect power(η^2^ = 0.045, 95%, CI 0.04–0.05). Post-hoc results showed that on average the possibilities to perform a *penetrative* pass had less time available than the opportunities to perform a *backward* pass, which in turn had less time available than the opportunities to perform a *support* pass.

The ANOVA analysis highlighted a significant effect of the factor Game on the mean passing availability (F_(4, 16132)_ = 28.07, *p* < 0.001; *1st:* 1.34 ± 1.13 s, *2nd:* 1.15 ± 1.04 s, *3rd:* 1.21 ± 1.18 s, 4th: 1.17 ± 1.17 s and 5th: 1.16 ± 1.15 s), with a low power effect (η^2^ = 0.007, 95%, CI 0.004–0.009), with the post-hoc analysis revealing that the first match had potential passes available for longer periods, while the remaining matches did not differ between them.

Additionally, there was a significant effect of the interaction between the two factors (F_(8, 16132)_ = 5.06, *p* < 0.001), with a low power of the effect (η^2^ = 0.003, 95%, CI 0.001–0.004). A post hoc analysis showed that *penetrative* and *backward* passing opportunities were available for longer periods in the first match than in the other four matches. Furthermore, the difference between *backward* and *penetrative* passes was only significant over the three first matches. Finally, *support* passing opportunities were similar between all five matches. Table 1 displays the exact time the passing opportunities were available per match, and type of pass.

## Discussion

Visually inspecting the obtained landscapes enables the interpretation of game patterns that may characterize a team’s performance. For example, we may suggest that the team under analysis had difficulties to create opportunities to pass within the opposing midfield, since most of passing opportunities were created in its own midfield. Specifically, *backward* passing opportunities were more spread over the field than the other two types of passes that are concentrated on a certain area of their own side of the field (expressed by the warmer colors in Fig. 2, left and middle columns). This supports the idea that within a football match, the set of (game) constraints^29^ influence the dynamics of player’s co-positioning. Consequently, there are some areas of a football field that are over-used compared to others, suggesting that opportunities for action and the threat that those actions may cause (e.g., a *penetrative* pass) are not homogeneously spread across the space.

Concerning heatmaps similarity, EMD results revealed that most matches displayed quite similar landscape patterns of passing opportunities, which means that the player’s spatial configuration of the team under analysis was able to maintain similar patterns of passing opportunities. For instance, landscapes of *backward* passing opportunities displayed the highest similarity between the five matches under analysis. A similar result was achieved for the first four matches regarding the *penetrative* and *support* passes opportunities. However, the fifth match demands a more detailed explanation. On the 5th match the EMD results displayed a low similarity of *penetrative* and *support* passing opportunities when compared with the remaining four (please see Fig. 4). Perhaps these results were due to the opponent team adopting a high defensive pressure strategy^30^ which decreases the space available to create these passing opportunities. Consequently, the heatmaps display a high concentration of *penetrative* and *support* passing opportunities in a short area of the field (please see Fig. 2 left and middle columns, bottom heatmap) which may led to a different landscape.

However, characterizing a football match in terms of passing opportunities with a single heatmap that covers the whole game dismisses the variation in dynamics that occur throughout a match (identical to the smoothing effect of temporal averaging). The analysis of the heatmaps with shorter temporal windows may enable to identify changes in the landscapes of passing opportunities that emerge during the course of a match and how they evolve with time. For example, in the 4th match it was possible to observe the transitions of *penetrative* passing opportunities from a spread area in the midfield to one area where the passing opportunities concentrated in the right side of their own midfield. As the first half ends, passing opportunities drastically drop, as opportunities for penetrative passes vanish in most areas of the field (please see Fig. 3). This example depicts transitions within the landscape of *penetrative* passing opportunities which allows to characterize the dynamics of the most threatening areas that arise along a football match.

Regarding the mean time that passing opportunities were available, there were differences over the matches and for the different type of passing opportunities. The first match had longer mean times for their potential passes than the other four (that did not differ between them). This is probably due to an opposing player receiving a red card on the 63^rd^ minute, which means that the opponent team played with one player less for approximately half an hour, which demands an adjustment on his team spatio-temporal configuration. Consequently, these adjustments may create additional difficulties to the team to defend and close passing opportunities but also to regain ball possession and create passing opportunities. Thus, this landscape model reinforces the notion that disturbances in a team initial configuration (e.g., a sent off player) alter players interactive behavior which consequently lead to changes in the time that a passing opportunities were available. It is noteworthy that this difference only happens for *backward* and *penetrative* passes and not for *support* passes, which suggests that these type of passing opportunities were less affected by the team defense capabilities and strategies.

Concerning *penetrative* passing opportunities been available for less time than *support* or *backward* passes (for the first three matches); these may be because *penetrative* passes will place the ball onto an off-ball player that is outplaying more opponents than the ball carrier, which is hypothetically more dangerous for the defending team than a *support* or a *backward* pass^3^. This theoretical threat may increase the defenders’ willingness to intercept passing opportunities, which consequently decreases the time that *penetrative* passing are available compared to *backward* or *support* passes. However, this should mean that *support* passing opportunities had less time available than *backward* passes. Nevertheless, the results revealed otherwise, which could be explain by a possible increased pressure to recover ball possession close to the opposing goal^7,31^. This could explain the results of the fourth and fifth match where there was no significant differences between the time that *backward* and *penetrative* passing opportunities were available, possibly due to a high defensive pressure of the opposing team.

Differences in the time that passing opportunities were available as well as visual differences in the landscapes of passing opportunities accordingly with the different type of passes, led us to suggest that this landscape model is sensible to changes in game constrains, such as defensive pressure of the opposing team, a red card shown to an opposing player, or other events that may change players spatio-temporal configuration allowing to identify the most vulnerable areas of the field. From an applied perspective, this landscape model provides relevant information for performance analysis and team technical staff about the passing opportunities that have been created by both teams due to the interactive behaviour among ball carrier and off-ball players (i.e., potential receivers and opponent players) as well as in which areas of the field and for how long those passing opportunities were available.

Regardless of the promising results, this model suggests three issues for further research: (1) so far the model assumed that all the players are technically and tactically equal. An issue for further research is to add individual technical characteristics to shape each player passing opportunities on a competitive match; (2) the outplay principle to categorize passes requires an accuracy improvement. *Penetrative* passes are not the only that can increase the probability of a goal as *backward* passes can also help in scoring goals^32^. This is especially relevant close to the opposing goal, where a pass *backwards* that creates a better angle to score could be a better decision than *penetrative* passes that leave the receivers in a worst angle to shoot to the goal. In this regard, it is important to keep in mind that previous research have shown that the location of the goal was a relevant task constrain in Futsal^33^; (3) currently a constant ball speed was assumed. As ‘real’ balls do not fly with constant speed, this could be leading to under or overestimation of the passing opportunities available and should therefore be reformulated (please see^34^, for a possible solution).

Based on ball carrier and off-ball players spatiotemporal interactions this landscape model depict the passing opportunities that emerge in the course of a football match. The landscapes of passing opportunities varied through different matches, especially for *support* and *penetrative* passes. Hence we may conclude that this model is sensitive to task constrains and to the uncertainty related with the uniqueness of each football match, at least regarding the landscape of passing opportunities. Nevertheless, it was possible to show evidence that due to player’s interactive behaviour some matches exhibit more similarity than others, as displayed by the EMD results.

Having the heatmaps as an output provides visual information regarding the areas of the field where more passing opportunities were available and for how long they last. Additionally by creating temporal heatmaps it was possible to identify how the threat (for the defense) caused by the different types of passing opportunities fluctuates in space and time during the course of a match. As expected, the different type of passing opportunities were available for different time windows. For different reasons *penetrative* and *backward* passing opportunities last less than *support* passing opportunities.

Finally, we reinforce that this is a methodological driven research, thus we do not aim for a generalization of results, but rather a generalization of the method usability. The outputs of this landscape model provides information that could be relevant to a post-match report shortening the gap between performance analysis team and the technical staff.

## Supplementary Information

Supplementary Video 1.

Supplementary Video 2.

Supplementary Video 3.
